# Precision prevention for neurodegenerative diseases: lessons learnt from Alzheimer’s disease translated into perspectives for Parkinson’s disease

**DOI:** 10.1007/s00702-025-03090-z

**Published:** 2025-12-26

**Authors:** Sibylle Béchet, Morgan Leonard, Esther Ademola, Joëlle V. Fritz, Rejko Krüger

**Affiliations:** 1 Luxembourg Institute of Health (LIH), Transversal Translational Medicine, 1A-B, Rue Thomas Edison, L-1445 Strassen, Luxembourg; 2 Translational Neuroscience, Luxembourg Centre for Systems Biomedicine (LCSB), University of Luxembourg, 6, Avenue du Swing, Belvaux, L-4367 Luxembourg, Luxembourg; 3 Parkinson Research Clinic, Centre Hospitalier de Luxembourg, 4, Rue Nicolas Ernest Barblé, 1210, Luxembourg, Luxembourg

**Keywords:** Neurodegenerative diseases, Prevention, Alzheimer’s disease, Parkinson’s disease, Dementia, Cognitive Impairment

## Abstract

Population ageing is posing significant challenges for healthcare systems and societies worldwide. Neurodegenerative diseases, including Alzheimer’s disease and Parkinson’s disease, are among the leading contributors to disability, dependence, and healthcare costs in ageing societies. Prevention offers the most sustainable approach to reducing the burden of neurodegenerative diseases. While Alzheimer’s disease prevention is already advancing through biomarker-based early detection, identification of modifiable risk factors, and multi-domain interventions, efforts are now turning towards Parkinson’s disease, the fastest growing neurodegenerative disorder. Using deeply phenotyped cohorts of patients with Parkinson’s disease and people with prodromal stages of neurodegenerative diseases, novel biomarkers have enabled biological classification for improved diagnosis of Parkinson’s disease, including early cognitive impairments. The expanding knowledge of Parkinson’s disease risk factors is now being translated into primary and secondary prevention concepts within integrated care settings to effectively address the burden of neurodegenerative diseases for the people affected. Integrating biomarker-based risk stratification with scalable life-style based programmes offers a realistic pathway toward precision prevention in Parkinson’s disease.

## Introduction

Based on improved medical care, populations are ageing worldwide, leading to an increase in the burden of chronic neurodegenerative diseases (Wang et al. 2024). Among these, Parkinson’s disease (PD) represents the fastest-growing neurological disorder in terms of prevalence, accompanied by parallel disability and death (Dorsey and Bloem 2018). The prevalence of the disease has doubled in the last 25 years, with further increase projected until the year 2050 (Su et al. 2025). Recent epidemiological analyses confirm that PD prevalence continues to rise globally, although incidence trends vary across regions, remaining stable or declining in some high-income countries while increasing elsewhere (Fink et al. 2025; Pereira et al. 2024). These regional differences potentially reflect differential environmental exposures, healthcare access, and lifestyle patterns, underscoring the potential influence of modifiable factors in shaping disease burden. Together, the growing prevalence and its consequences emphasise the scale of the public health and socioeconomic challenge posed by PD. Epidemiological evidence accentuates both, the substantial burden of disease and its heterogeneity in presentation, progression, and clinical outcomes (de Lau and Breteler 2006; Höglinger et al. 2024). Characterizing PD as a pandemic highlights its growing scale and urgency, reinforcing the necessity for coordinated global responses comparable to those mobilized against other major health crises (Dorsey and Bloem 2018). Similar discussions have occurred earlier in the Alzheimer’s field, where the recognition of dementia as a global challenge has mobilised substantial international efforts. The *World Alzheimer Report 2024*, highlights shifting societal attitudes toward dementia and strengthens the necessity of prevention-oriented strategies (World Alzheimer International 2024). This growing recognition that neurodegenerative diseases can be shaped by modifiable risk factors has therefore established a prevention framework increasingly applicable to PD. In dementia research, landmark analyses have demonstrated that a substantial proportion of cases could be prevented or delayed through active modification of lifestyle (Livingston et al. 2024; Mukadam et al. 2024). Building on this concept, recent work suggests that similar multi-domain prevention strategies may also be relevant for mitigating cognitive decline in PD (Kalbe et al. 2025a, b).

Recent advances in the understanding of the molecular underpinnings of neurodegenerative diseases, including the identification of biomarkers derived from cerebrospinal fluid (CSF), revealed that the neurodegenerative process underlying e.g. in Alzheimer’s disease can be detected even years before first clinical symptoms present. Based on this observation, for Alzheimer’s disease, clinical–biological frameworks have been proposed to classify individuals based on biomarkers and disease biology, independent of clinical stage (Jack et al. 2018; Jia et al. 2024).

Similar to AD, recently a first concept for a biomarker in PD was established based on aggregated forms of α-synuclein. Initially, α-synuclein was established as the first genetic cause of PD based on linkage analyses in rare families with inheritance of the disease and the subsequent identification of point mutations changing the amino acid sequence conferring a toxic gain of function(Polymeropoulos et al. 1997; Krüger et al. 1998). Subsequently the α-synuclein protein was identified as the main component of the pathognomonic Lewy bodies in brains of sporadic idiopathic PD patients (Spillantini et al. 1997), indicating a more general role of the protein in PD pathogenesis. Additional point mutations in the *SNCA* gene (e.g., E46K, G51D) as well as duplications and triplications of the *SNCA* locus were identified and pathological α-synuclein aggregation in the respective mutation carriers, establishing α-synuclein as both a genetic driver and pathological hallmark of the disease genetic and idiopathic PD (Oliveira et al. 2021). Importantly, first association studies revealing a role of common genetic variants in the SNCA gene with sporadic forms of PD were later confirmed in large cohorts world-wide and established SNCA as the most common risk factor for PD across different ethnicities (Krüger et al. 1998; Simon-Sanchez et al. 2009). The implementation of α-synuclein seed aggregation assays from CSF and more recently in blood of people with typical sporadic PD has enabled a first biological staging system that account for synuclein pathology, neurodegeneration and genetics (Höglinger et al. 2024; Okuzumi et al. 2023; Orru et al. 2025; Simuni et al. 2024). First studies in people with REM-sleep behavioural disorders (RBD) as a prodromal stage of PD using α-synuclein seed amplification assays demonstrated that biomarkers may be detected years before the clinical onset of motor symptoms of PD, opening a window for preventive interventions (Concha-Marambio et al. 2023; Siderowf et al. 2023). In addition to RBD, other prodromal signs, such as hyposmia, constipation and autonomic dysfunction may manifest years before motor onset of PD, providing further opportunities for early detection and disease-modifying interventions (Poewe et al. 2017). Together, these developments suggest a paradigm shift: from considering PD and related conditions as inevitable consequences of a neurodegenerative process related to ageing to considering them, at least in part, as preventable disorders by acting on modifiable risk factors. Building on experiences from Alzheimer’s prevention research, there is now a critical opportunity to develop preventive strategies and find ways to implement them for people with PD with broad access, particularly in well-established integrated care concepts (Bloem et al. 2017; Schröder et al. 2024).

### The need for prevention in neurodegenerative diseases

**Fig. 1:**
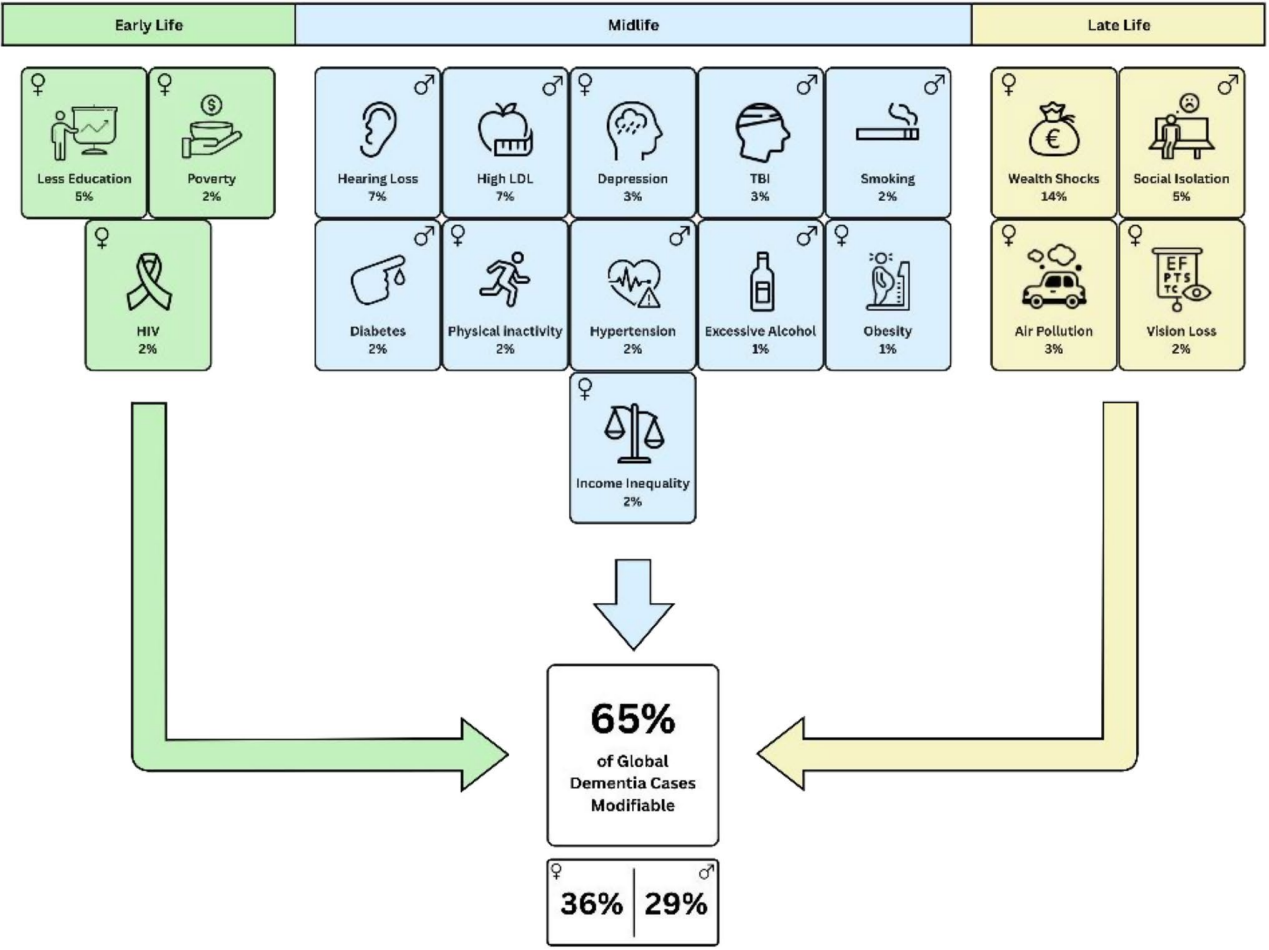
Modifiable risk factors for dementia across the lifespan (modified from expanded model by Mostert et al. (2025)). Each factor is shown with its estimated contribution to global dementia, indicating the percentage reduction in cases if this factor was eliminated. Icons denoting sex indicate higher prevalence of this factor in that sex group. Also denoted is potential modifiable risk separated by female-and male-specific factors. *HIV* human immunodeficiency virus, *LDL* low density lipoprotein cholesterol, *TBI* traumatic brain injury

**Fig. 2:**
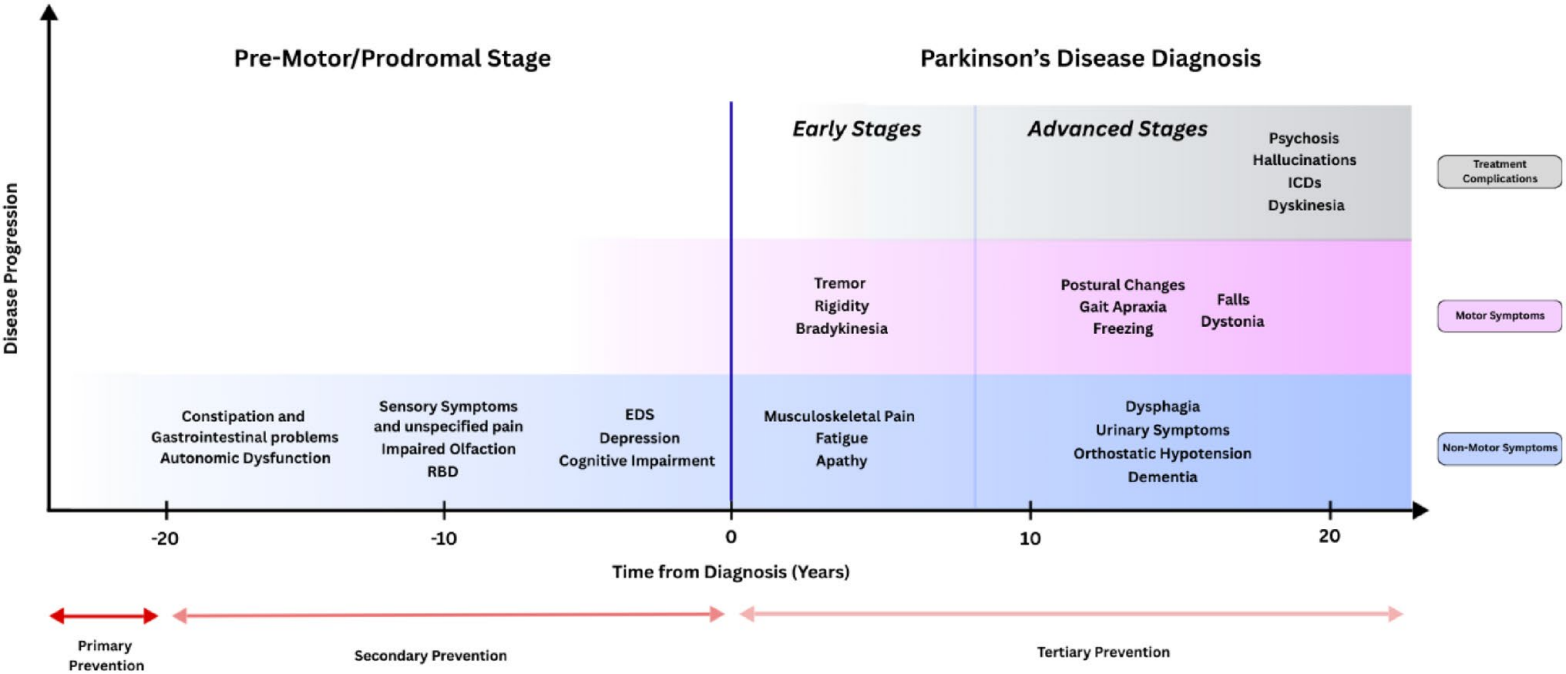
Chronological progression of Parkinson’s Disease symptomatology (adapted from Castilla Cortázar et al. (2020)). The illustration represents typical emergence and progression of symptoms from the prodromal phase through early and advanced stages of the disease, spanning approximately 20 years before and after clinical diagnosis. Motor and non-motor features, as well as treatment induced complications are depicted across disease progression. Windows for primary, secondary, and tertiary prevention strategies are also shown. *RBD* REM-sleep behaviour disorder, *EDS* excessive daytime sleepiness, *ICDs* impulse control disorders

Neurodegenerative diseases develop over decades before symptoms emerge (Katsuno et al. 2018). This long prodromal phase offers a crucial opportunity for preventive action. In both AD and PD, early pathological processes precede clinical manifestation by many years. In Alzheimer’s disease, early pathological changes are reflected by biomarkers in the CSF and, more recently established, in blood. Decreases in amyloid-β indicate amyloid deposition (A), increases in phosphorylated and total tau reflect tau pathology (T), and markers such as elevated neurofilament light chain capture neurodegeneration (N), which correlates with cortical and hippocampal atrophy in the MRI (Jack et al. 2024; Jia et al. 2024). These biological changes emerge up to two decades before the onset of cognitive symptoms (Jia et al. 2024). Corresponding approaches are now emerging for PD, where aggregation of α-synuclein can be detected in CSF and blood using seed amplification assays (Concha-Marambio et al. 2023; Okuzumi et al. 2023). Here, high sensitivity and specificity were demonstrated for detecting PD cases compared to controls and first evidence for the presence of pathological α-synuclein aggregation in prodromal stages of PD was shown (Okuzumi et al. 2023; Simuni et al. 2024). Yet, longitudinal confirmation still is required in these prodromal cases to determine sensitivity and specificity of seed amplification assays for identifying individuals at risk of developing PD (Figs. 1, 2).

This perspective underpins the framework of primary, secondary, and tertiary prevention. Primary prevention seeks to reduce incidence by proactively addressing lifestyle and environmental risk factors before onset of the disease process. Secondary prevention targets at-risk individuals identified through biomarkers or genetic profiling with an ongoing neurodegenerative process, aiming to delay clinical onset. This represents a conceptual shift – while prevention was traditionally defined by the onset of clinical symptoms, emerging biomarker-based frameworks now allow identification of at-risk but asymptomatic individuals. Although this approach is not yet applied in clinical practice, it offers the prospect of earlier, biology-driven classification and subsequent preventive strategies. Tertiary prevention focuses on minimising the disease impact in patients by slowing progression and avoiding complications after diagnosis. In neurodegeneration, the long prodromal phase makes secondary prevention particularly promising as it may delay the neurodegenerative process to an extent, where the full clinical syndrome may not be experienced during lifetime. Evidence from dementia research further illustrates the potential impact of such an approach. Careful scientific analyses of available studies as performed by the Lancet Commission on Dementia Prevention have estimated that up to 45% of dementia cases worldwide could be prevented or delayed by modifying key risk factors across lifespan (Livingston et al. 2024).

Cognitive impairment is also a major feature of PD trajectory, with mild cognitive impairment (MCI) being already present in approximately 25–50% of patients at the time of diagnosis, and longitudinal data indicating that about one-third to 40% develop dementia over course of their disease (Gallagher et al. 2024). Despite this, the World Alzheimer Report (2024) reveals persistent misconceptions: nearly 80% of the public and 65% of healthcare professionals still consider dementia as a normal part of ageing, and more than a quarter believe nothing can be done to prevent it (Alzheimer’s Disease International, 2024). Such misconceptions not only perpetuate stigma but also hinder timely engagement in prevention strategies.

### Lessons from dementia prevention

Dementia,particularly Alzheimer’s disease, has become a model case for prevention-oriented research in neurodegeneration, illustrating how both biological insights as well as public health initiatives can be combined to reduce disease burden. One of the most significant developments has been the shift from clinical toward biological diagnostic frameworks, such as the National Institute on Aging and Alzheimer’s Association (NIA-AA) criteria and the International Working Group recommendations, which define AD not only by its clinical symptoms but also by its biological signature (Dubois et al. 2024; Jack et al. 2018). These approaches, grounded in biomarker evidence, redefined AD as a biological construct that can be identified years before clinical symptoms appear. This prodromal phase, encompassing subjective cognitive decline (SCD) and MCI, represents a critical window for preventive intervention, shifting the focus from symptomatic treatment to risk reduction in at-risk but still independent individuals.

In terms of public health strategies, dementia research has also highlighted the importance of modifiable risk factors. The *Lancet Commission on Dementia Prevention, Intervention and Care* (2020; updated 2024) concluded that up to 45% of dementia cases worldwide could be prevented or delayed by addressing modifiable risk factors across the lifespan. The Commission initially identified 12 modifiable risk factors and now recognises 14, including physical inactivity, hypertension, diabetes, obesity, depression, hearing loss, social isolation and air pollution, underscoring the growing evidence for dementia prevention (Livingston et al. 2024).

One of the lessons from dementia prevention research is that multi-domain lifestyle interventions can preserve cognitive function in at-risk populations. The landmark Finnish Geriatric Intervention Study to Prevent Cognitive Impairment and Disability (FINGER) demonstrated that a two-year program combining exercise, dietary guidance, cognitive training, and vascular risk factor management stabilized cognitive performance in older adults compared to standard care (Ngandu et al. 2015). Subgroup analyses further showed that even individuals with high genetic risk, such as APOE ε4 carriers, benefitted, underscoring the broad applicability of lifestyle-based prevention. Building on this success, the World-Wide FINGERS consortium is now adapting and replicating the model internationally. Early reports indicate that multi-domain interventions can be feasibly implemented across diverse healthcare systems and cultural contexts, with full outcome results expected from ongoing trials (Kivipelto et al. 2020; Rosenberg et al. 2020). More recently, the U.S POINTER two-year randomised clinical trial enrolled over 2000 older adults at risk of developing cognitive decline and dementia. The study found that a structured multi-domain lifestyle intervention involving increased physical and cognitive activity, healthy diet, social engagement, and cardiovascular health monitoring resulted in significant greater improvement in global cognition over 2 years relative to a lower intensity self-guided intervention (Baker et al. 2025).

Beyond trials, epidemiological studies provide complementary evidence. In several high-income countries, dementia incidence has shown signs of decline, a trend attributed to better education and improved management of vascular risk factors (Mukadam et al. 2024; Satizabal et al. 2016). Microsimulation models further suggest that if these trends continue, the future prevalence of dementia may be lower than earlier projections (Bruck et al. 2022). By contrast, in many low- and middle-income countries, dementia incidence has not declined and rising prevalence of risk factors such as diabetes, hypertension and obesity may offset some of the gains seen elsewhere (Norton et al. 2014; Wu et al. 2017). Together, these studies suggest that prevention strategies can alter the course of disease burden on a population level, although benefits remain unevenly distributed across different socioeconomic groups.

Scientific evidence for AD and dementia prevention is strong, yet its benefits are not yet evenly distributed across populations. As highlighted in the World Alzheimer Report (2024), persistent misconceptions remain a barrier to early engagement, reinforcing the lesson that effective prevention requires not only identifying interventions but also communication and education strategies to provide access to these measures and to ensure that individuals and healthcare systems recognise prevention as possible and worthwhile. AD experience shows that public awareness campaigns, training of healthcare professionals, and consistent messaging to carers are needed to translate scientific advances into behavioural change and early engagement (World Alzheimer International 2024; Livingston et al. 2024). Together, dementia prevention research highlights that early, targeted interventions can modify disease trajectories, providing a foundation for extending similar prevention concepts to PD, where opportunities for early detection and risk reduction are now beginning to emerge.

### Parkinson’s disease: the next frontier in prevention

Building on advances from AD and dementia research, growing attention is now being directed toward prevention in PD. Such efforts have only recently become feasible, as the prodromal stages of PD and their biological underpinnings are being elucidated, most notably through identification of at-risk individuals (e.g. RBD patients) and implementation of α-synuclein seed amplification assays, which allow the detection of synuclein pathology already in these prodromal stages of the disease (Okuzumi et al. 2023). In the absence of disease-modifying pharmacological treatments, prevention has gained even greater importance for PD. Current research therefore focuses on identifying at-risk individuals and implementing strategies that may reduce risk, delay onset, or slow progression through lifestyle based approaches.

Physical activity represents the strongest evidence base for PD prevention. Epidemiological studies show that regular aerobic exercise is associated with reduced risk of developing PD, supporting its role in primary prevention (Lin et al. 2024; Paillard et al. 2015). Large cohort studies further show that lifestyle factors such as lower body mass index and leisure-time physical activity are linked to a reduced risk of PD, with even moderate exercise conferring measurable benefit (Sääksjarvi et al. 2014; Yang et al. 2015). In already diagnosed PD patients, clinical and brain imaging studies demonstrated that structured exercise can stabilise or even restore functional brain networks (Johansson et al. 2022), thus highlighting its value for secondary prevention and disease management.

Beyond physical activity, other lifestyle domains also contribute to risk modification. While diet-related risk reduction is well-established in dementia prevention, corresponding evidence in PD is emerging and points toward converging mechanisms across neurodegenerative conditions. Dietary factors have drawn particular attention, with adherence to a Mediterranean diet associated with a lower risk of PD (Maraki et al. 2023; Zhao et al. 2025). Furthermore, epidemiological evidence links type 2 diabetes as well as pre-diabetes to an increased risk of PD, with impaired insulin signalling and chronic inflammation contributing to dopaminergic vulnerability (Chohan et al. 2021; Sanchez-Gomez et al. 2021; Yu et al. 2022). Experimental data further highlights the role of insulin resistance in PD pathogenesis, particularly in GBA1-associated PD, where impaired insulin signalling exacerbates dopaminergic neuron loss in vitro (Zagare et al. 2025). These findings suggest that strategies to improve insulin sensitivity, including dietary modification and metabolic interventions, may have preventative potential.

Mild cognitive impairment is already present in approximately 19–36% of PD patients at the time of diagnosis, with deficits across attention, memory and executive function in newly diagnosed PD (Aarsland et al. 2009; Baiano et al. 2020; Muslimovic et al. 2005; Yarnall et al. 2014). A recent systematic review and meta-analysis estimated that about 4.5% of people with PD develop dementia each year, representing a threefold higher risk compared with healthy controls (Gibson et al. 2024). Importantly, up to 83% of people with PD develop dementia at 20 years of diagnosis (Aarsland et al. 2021). Thus, although cross-sectional cohorts typically report cognitive impairment in around 40% of patients, the cumulative risk of cognitive impairment or dementia over the disease course is substantially higher. Additionally, the concept of subjective cognitive decline (SCD) increasingly attracts attention in the context of PD. While initially described in the context of AD, SCD describes impairment that is subjectively perceived by the patient, but which is not clinically detectable using neuropsychological assessments. Increasing evidence indicates that SCD precedes MCI in people with PD, with a relative risk of cognitive decline of 2.71 within an average follow-up period of 3.16 years (Siciliano et al. 2024). These observations underline that cognitive changes emerge early in PD, suggesting that protective factors could delay deterioration. The concepts of cognitive reserve and lifestyle enrichment, initially introduced in the context of AD, have increasingly gained attention for PD (Kalbe et al. 2025b). Individuals with higher levels of education, mental activity, and engagement in cognitively stimulating pursuits show better outcomes and may be more resilient to neurodegeneration (Hindle et al. 2016; Muslimovic et al. 2005).

Social factors are increasingly recognised as well; both Geng et al. (2024) and Terracciano et al. (2023) reported that social isolation is linked to an elevated risk of PD, independent of demographic, socioeconomic, or genetic factors. Collectively, these findings highlight the potential for multidimensional lifestyle approaches in PD prevention and mirror the patterns observed in dementia research. Together, they point toward shared mechanisms across neurodegenerative diseases, suggesting that lifestyle and metabolic factors shape an across-dementia prevention phenotype that is accessible to intervention.

Beyond lifestyle approaches, progress in pharmacological prevention depends on demonstrating effective disease modification in clinically manifest PD, as recently shown for Alzheimer disease with the modest effects of lecanemab and donanemab, antibodies raised against pathological beta-amyloid, in clinical trials slowing down the progression of cognitive decline (Sims et al. 2023; van Dyck et al. 2023). While recent trials of α-synuclein–targeted therapies, such as prasinezumab, have shown encouraging signals, including delayed motor progression (Nikolcheva et al. 2025; Pagano et al. 2024), definitive evidence for disease-modifying efficacy is still pending. Once such effects are confirmed, the next step will be to evaluate the potential of these compounds earlier in the disease course, i.e. during prodromal stages like iRBD, where they may have the potential to prevent the occurrence of motor symptoms. Success in this domain relies on well-characterized and stratified PD and at-risk cohorts, which provide the necessary platform to test disease-modifying interventions. PD is highly heterogeneous, with subgroups defined by genetic background, biomarker profiles, and clinical phenotypes (Hu et al. 2024). Identifying and characterising at-risk cohorts is essential for tailoring prevention strategies, designing targeted interventions, and facilitate clinical trials that intervene before irreversible neurodegeneration occurs. Therefore, international efforts now aim at establishing deep-phenotyped large cohorts of at-risk individuals, like the multi-centre Healthy Brain Ageing Consortium, which allows to detect early stages of neurodegenerative diseases and identification of individual trajectories (Schade et al. 2025). These cohorts will enable robust stratification into trial-ready subpopulations for preventative pharmacological treatments along intelligent clinical trials designs improving feasibility and cost-effectiveness of drug development.

Genetic discoveries over the last two decades have made up to 30% of cases with PD explainable by monogenic forms, e.g. LRRK2 as the most common autosomal-dominant form of PD or relevant disease-associated variants, e.g. GBA1 (Zimprich et al. 2004; Neumann et al. 2009). Importantly, these genetic forms served as prototypes to study the pathogenesis for also the more common sporadic form of PD and thereby yielded directly targetable pathways for precision medicine in PD. LRRK2 mutations, particularly G2019S, represent the most common LRRK2 variant in the western populations, and have spurred the development of selective LRRK2 kinase inhibitors (Pena et al. 2024). Pre-clinical and early clinical data show that both type I and type II inhibitors can suppress pathogenic kinase activity, improve lysosomal and vesicular trafficking, and modulate Rab GTPase phosphorylation (Karami et al. 2025; Raig et al. 2025). Despite some promising preclinical results, LRRK2-targeted therapies remain limited by concerns about long-term safety, particularly pulmonary and renal morphological changes observed in toxicology studies (Karami et al. 2025). The discontinuation of the Phase 3 LIGHTHOUSE study of BIIB122 (DNL151) (NCT05418673) further illustrates the developmental challenges in this therapeutic area, even though its termination was driven by strategic considerations rather than reported toxicity. More recently, variants in RAB32 have been identified as novel genetic risk factors for PD and shown to interact functionally with LRRK2-dependent pathways. This underscores the convergence of vesicular and mitochondrial trafficking mechanisms in genetically stratified forms of the disease (Gustavsson et al. 2024) and may indicate stratification options for precision medicine, as Rab32 is acting downstream of LRRK2 (Zimprich et al. 2004; Gustavsson et al. 2024). Recently, PRKN, the most frequent cause of autosomal-recessive early-onset PD, has emerged as a therapeutic target through its central role in ubiquitination and mitophagy, supporting strategies that enhance parkin function to improve mitochondrial function and clearance in this defined genetic subgroups (Safreena et al. 2024; Clausen et al. 2024; Senkevich et al. 2022). Interestingly, polygenic risk scores focusing on genes involved in mitochondrial homeostasis were not only confirmed to be associated with subtypes of sporadic PD in different cohorts but have been functionally validated in neuronal cells derived from induced pluripotent stem cells of PD patients carrying a high-risk profile (Arena et al. 2024). Together, these targets exemplify how genetics can delineate at-risk populations and provide mechanistically grounded entry points for evaluating disease-modifying interventions.

## Discussion

Over the past decade, the understanding of neurodegenerative diseases has shifted from conditions defined by their clinical symptoms to biological entities identifiable long before the onset of measurable cognitive or motor disability. This transformation opens a crucial window for early interventions, but it also raises ethical and conceptual challenges about how to act on biological risk: biomarker-positive but still asymptomatic individuals may be labelled as “patients-in-waiting” despite uncertain clinical progression. Among these conditions, PD represents a particularly urgent case as the number of people affected by this condition is growing fast worldwide (Meissner et al. 2024). Despite major advances in understanding of the underlying molecular mechanisms, no disease-modifying treatment currently exists, and pharmacological management remains largely symptomatic. These limitations place renewed emphasis on prevention as a means to increase quality of life of people at risk and reduce disease burden for society. Prevention in PD can be envisioned as a continuum—ranging from lifestyle and population-level approaches to biomarker-guided and pharmacological strategies. As lessons from dementia prevention have shown, identifying modifiable risk factors and targeting prodromal stages such as REM sleep behaviour disorder, hyposmia, or autonomic dysfunction can open opportunities for effective intervention long before clinical onset.

While the possibility of detecting α-synuclein pathology in prodromal stages of PD such as iRBD represents a major advance, it also raises conceptual and ethical considerations regarding prevention staging and communication. Interventions in biomarker-positive individuals represent secondary prevention, as the neurodegenerative process may be already active despite absence of clinical manifestations. These individuals should therefore be described as “at risk” rather than diagnosed with PD. Still communication of risk profiles may support motivation for adherence to lifestyle-based interventions, which depends on perceiving preventive measures as empowering and purposeful rather than as treatment for inevitable decline. Importantly, prevention frameworks continueto evolve as new evidence constantly emerges (Baker et al. 2025). The Lancet Commission on dementia prevention, intervention and care has recently expanded its list of modifiable risk factors from 12 to 14, reflecting the growing evidence base for factors such as air pollution and traumatic brain injury (Livingston et al. 2024). Complementary analyses further highlight the contribution of broader social determinants and influences of sex and gender dimensions to dementia risk (Mostert et al. 2025). Emerging data also point to the relevance of sleep quality and circadian regulation as additional determinants of neurodegenerative risk. These ongoing updates illustrate that prevention is a dynamic, continuously refined field, integrating new biological, behavioural, and environmental insights as they arise.

### How to provide access: Luxembourg as a model for integrated precision prevention

Luxembourg provides an instructive example of how precision prevention for neurodegenerative diseases can already be effectively implemented within a public healthcare system on the national level and benefit from close interaction with excellent clinical and fundamental research (Schröder et al. 2024) (https://parkinson.lu/). While Luxembourg benefits from a well-resourced health system (European Observatory on Health Systems and Policies 2024), key components of its approach, such as population-based risk profiling, multi-domain lifestyle interventions, and scalable digital prevention tools, illustrate what is already feasible for neurodegeneration, even though such integrated prevention frameworks are not yet widely established for many other disease areas. Nevertheless, these elements can be adapted to diverse healthcare contexts, including lower-resource settings, where the burden of neurodegenerative diseases is rapidly increasing. For example, as hypertension has been widely associated with MCI and dementia globally (Stephan et al. 2024), decreased detection and unregulated monitoring of blood pressure may heighten the risk of cognitive decline in lower-income regions (Gao et al. 2009; Ogunniyi et al. 2011). One may then seek to educate non-medical professionals on the use of blood pressure monitors, thereby promoting nationwide accessibility and increasing efforts to modulate hypertension as a causal factor for cognitive decline and dementia. Through population-based cohorts such as the *Healthy Brain Ageing* (HeBA; https://heba.lu/en/) study and the *RBD* cohort (https://www.rbd.lu/en/), individuals at risk for neurodegenerative diseases are identified from the general population and studied longitudinally. These deeply phenotyped cohorts combine clinical, biomarker, and genetic profiling to define distinct at-risk subgroups and to map disease trajectories over time (Hipp et al. 2018; Landoulsi et al. 2023; Pachchek et al. 2023; Pavelka et al. 2023). Beyond their biological value, these cohorts also provide an opportunity to study early cognitive and behavioural markers of vulnerability. Recent findings underscore the importance of SCD as an early marker of vulnerability in alpha synucleinopathies (Ophey et al. 2025). In individuals with isolated RBD, SCD has been associated with subtle cognitive and structural brain changes, supporting its inclusion in longitudinal cohort studies aimed at identifying prodromal PD (Kalbe et al. 2025b; Ophey et al. 2025). Together, these data illustrate how Luxembourg’s cohort-based approach enables both the biological and societal dimensions of early detection to be studied within the same framework. The *HeBA* study, for instance, revealed that 79% of participants wished to know their individual risk for PD—even in the absence of available treatment (Mahlknecht et al. 2025). This finding underscores both societal readiness for prevention and the ethical responsibility to provide actionable pathways for those identified as at risk. Importantly, while pharmacological interventions are still emerging, structured preventive measures can already be offered today.

In order to be able to integrate most recent scientific evidence into scalable public health strategies for dementia in Luxembourg, the Programme Dementia Prevention (pdp) was launched in 2018 as one of the first nationwide frameworks for personalised dementia risk reduction (Schröder et al. 2024). The programme targets individuals with SCD or MCI, representing a stage of secondary prevention when early symptoms are present but functional independence remains. Participants are referred by their treating physician and undergo detailed neuropsychological assessment and multi-domain risk factor profiling. Based on these results, they receive vouchers that grant access to individually tailored lifestyle interventions—such as physical activity programmes, dietary counselling, or cognitive training. To date, over 700 individuals have been referred, with a mean age of approximately 68 years and balanced gender distribution, demonstrating both feasibility and public acceptance (Meissner et al. 2024).

Beyond its initial scope, the pdp has evolved into a broader public health initiative through digital and educational innovations. The *MyBraincoach* app, adapted from the Dutch *Breincoach*, extends the programme’s reach beyond clinical settings to the general population (https://www.mijnbreincoach.eu/) (Heger et al. 2019). It provides personalised feedback on modifiable lifestyle risk factors for dementia and PD, available in several languages to ensure accessibility across Luxembourg’s diverse population (MijnBreincoach, pdp). This initiative aligns with international campaigns such as the Dutch public health programme *“Wij zijn zelf het medicijn”* (*We Are the Medicine Ourselves*), which promotes awareness of brain-healthy lifestyles and underscores that prevention is both a medical and societal responsibility (Lgr imbu 2025). Together, these efforts demonstrate that dementia, and by extension, PD can be reframed as at least partly preventable conditions, empowering individuals to act on risk factors long before disease onset.

Luxembourg’s prevention strategy is strengthened further by its integrated care infrastructure, which directly embeds prevention within clinical and community practice. The *Réseau de Compétences Maladies Neurodégénératives*, launched in 2023, represents the first national integrated care network for neurodegenerative diseases and for the first time includes prevention as a structural component to multidisciplinary care networks. Built on the *ParkinsonNet* model established in the Netherlands (Meissner et al. 2024), this multidisciplinary network connects healthcare professionals across disciplines and care levels, ensuring equitable access to specialised care and promoting continuity between prevention, treatment, and rehabilitation. The alignment of these strategies with population-based research cohorts and digital prevention initiatives shows that it is possible, on the national level, to combine research, clinical care, and public health within a coherent and scalable framework. Current efforts in Luxembourg thus exemplify that identification of individuals at risk is warranted as effective prevention is not a distant ambition but a practical reality when research, healthcare, and policy are aligned toward a shared goal.

While lifestyle-based and behavioural interventions form the foundation of prevention today, pharmacological strategies represent the next frontier and are constantly evolving. Advances in biomarker discovery and genetic profiling now make it feasible to identify subgroups likely to benefit from targeted interventions before irreversible neuronal loss occurs. The *PreCoDe* trial (NCT07055087) exemplifies this concept of precision prevention, evaluating α-synuclein–directed therapy (prasinezumab) in GBA mutation carriers, an at-risk population identified through Luxembourg’s and partner cohorts. Such studies mark a conceptual shift from treatment to true disease interception. Similarly, repurposed metabolic drugs, including GLP-1 receptor agonists such as semaglutide and lixisenatide, are under active investigation in both AD and PD (Cummings et al. 2025; Meissner et al. 2024). These agents targetshared mechanisms, such as insulin resistance, mitochondrial dysfunction, and neuroinflammation, and may provide cross-disease benefits that bridge neurodegeneration and metabolic health (Hong et al. 2024), even if a final conclusion is still pending (Vijiaratnam et al. 2025). Such approaches will rely critically on accurate stratification, using genetic, biological and clinical profiling to identify individuals most likely to benefit from specific therapeutic interventions. Together, these developments suggest a future in which pharmacological and lifestyle-based prevention converge within stratified frameworks tailored to individual risk profiles.

Ultimately, PD prevention will rely on the interplay between early identification, behavioural modification, and biological intervention. Luxembourg’s example shows that these elements can coexist within a single framework: lifestyle-based prevention available now, precision pharmacological strategies on the horizon, and an integrated care system ready to deliver both. As prevention becomes the defining paradigm of neurodegenerative medicine, Luxembourg’s experience underscores a broader transformation—from reactive disease management to proactive maintenance of brain health across the lifespan.

## Data Availability

No datasets were generated or analysed during the current study.
